# Treatment of obesity-related diabetes: significance of thermogenic adipose tissue and targetable receptors

**DOI:** 10.3389/fphar.2023.1144918

**Published:** 2023-06-26

**Authors:** Ruping Pan, Jiadai Liu, Yong Chen

**Affiliations:** ^1^ Department of Nuclear Medicine, Tongji Hospital, Tongji Medical College, Huazhong University of Science and Technology, Wuhan, China; ^2^ Department of Endocrinology, Internal Medicine, Tongji Hospital, Tongji Medical College, Huazhong University of Science and Technology, Wuhan, China; ^3^ Laboratory of Endocrinology and Metabolism, Ministry of Education, Key Laboratory of Vascular Aging, Tongji Hospital, Tongji Medical College, Huazhong University of Science and Technology, Wuhan, China; ^4^ Branch of National Clinical Research Center for Metabolic Diseases, Wuhan, Hubei, China

**Keywords:** diabetes, obesity, brown adipose tissue, beige adipose tissue, receptor

## Abstract

Diabetes mellitus is mainly classified into four types according to its pathogenesis, of which type 2 diabetes mellitus (T2DM) has the highest incidence rate and is most relevant to obesity. It is characterized by high blood glucose, which is primarily due to insulin resistance in tissues that are responsible for glucose homeostasis (such as the liver, skeletal muscle, and white adipose tissue (WAT)) combined with insufficiency of insulin secretion from pancreatic β-cells. Treatment of diabetes, especially treatment of diabetic complications (such as diabetic nephropathy), remains problematic. Obesity is one of the main causes of insulin resistance, which, however, could potentially be treated by activating thermogenic adipose tissues, like brown and beige adipose tissues, because they convert energy into heat through non-shivering thermogenesis and contribute to metabolic homeostasis. In this review, we summarize the function of certain anti-diabetic medications with known thermogenic mechanisms and focus on various receptor signaling pathways, such as previously well-known and recently discovered ones that are involved in adipose tissue-mediated thermogenesis and could be potentially targeted to combat obesity and its associated diabetes, for a better understanding of the molecular mechanisms of non-shivering thermogenesis and the development of novel therapeutic interventions for obesity-related diabetes and potentially diabetic complications.

## 1 Introduction

Obesity is one of the main pathogeneses of type 2 diabetes mellitus (T2DM). The incidence of obesity and T2DM is increasing worldwide due to changes in human lifestyle and dietary structure. High glucose levels in blood circulation lead to dysfunction in the cardiovascular system, kidneys, nervous system, etc. Diabetic nephropathy is one of the principal causes of diabetic mortality. Obesity-related diabetes is largely attributed to the development of insulin resistance, which is primarily caused by “unhealthy” white adipose tissue (WAT) expansion and pro-inflammatory macrophage-mediated WAT inflammation (Odegaard et al., 2007). Additionally, insufficient insulin secretion from pancreatic β-cells is another important cause of T2DM. Reasons for pancreatic β-cell dysfunction in T2DM patients may include the accumulation of lipid droplets and dysfunctional mitochondria in these cells (Horii et al., 2022; Aoyagi et al., 2023).

Except for diet control and kinesitherapy, which are difficult to sustain, therapeutic strategies for the treatment of T2DM include increasing the sensitivity of target cells to insulin [sulfonylureas and thiazolidinediones (TZDs)/peroxisome proliferator-activated receptor γ (PPARγ) activators], moderate glucose levels either by increasing glucose uptake and usage in peripheral tissues such as skeletal muscle (metformin) or by inhibiting α-glucosidase in intestinal mucosal epithelial cells to reduce glucose uptake (α-glucosidase inhibitors), inhibit glucose reabsorption through renal tubules [sodium-glucose co-transporter 2 (SGLT2) inhibitors], and facilitate insulin secretion from pancreatic β-cells [dipeptidyl peptidase 4 (DPP4) inhibitors, glucagon-like peptide-1 receptor (GLP-1R) agonists, and amphetamine-acid derivatives] and insulin treatment. Drug safety profiles are certainly a big concern. Early-developed drugs, such as sulfonylureas, show a high risk of hypoglycemia and weight gain and may be associated with a hyperosmolar hyperglycemic state or pancreatic dysfunction (Pfeifer et al., 1984; Stoner et al., 2005). Although used as a first-line therapy in the past and present, the main concern with metformin is its gastrointestinal side effects (Florez et al., 2010). Off-target effects of several drugs, such as TZDs and DPP4 inhibitors, are also reported, and some are even fatal; for example, TZDs could cause hepatotoxicity, myocardial infarction, bladder cancer, and even liver/heart failure (Nesto et al., 2003; Rogue et al., 2010; Lewis et al., 2011), while DPP4 inhibitors often lead to upper respiratory tract infections and nasopharyngitis (Monami et al., 2011; Karagiannis et al., 2012). Due to the renal excretion of most of the drugs, renal impairment during anti-diabetic treatment is always under observation, and most of them, including SGLT2 inhibitors, are even prohibited for use in patients with severe renal dysfunction (Guthrie, 2012). Primarily, weight control is the most fundamental treatment for T2DM. Among the aforementioned therapeutic interventions, GLP-1R agonism alternatively seems to be an anti-obesity treatment, as GLP-1R agonists work as potent extrinsic appetite suppressants. Certain GLP-1R agonists could penetrate through the blood–brain barrier and probably act on the hypothalamus to increase central satiety on the one hand (Seufert and Gallwitz, 2014; Blundell et al., 2017), and on the other hand, they could act on the gastrointestinal tract to delay gastric emptying (Friedrichsen et al., 2021). Probably, GLP-1R agonism could be a competent therapeutic strategy to combat obesity-related diabetes with minimal side effects (such as nausea and vomiting), also because of its beneficial effects on the nervous and cardiovascular systems and metabolic equilibrium (Zhao et al., 2021). Interestingly, the antiatherogenic benefit of GLP-1R agonists is independent of GLP-1Rs in either endothelial cells or hematopoietic cells, whereas the inhibitory effects of GLP-1R agonists on cytokine expression, triglyceride accumulation, and fibrosis in the high-fat, high-cholesterol diet-fed mouse liver require GLP-1R expression in the cells targeted by Tie2-Cre (possibly T cells) (McLean et al., 2021). However, the invasive subcutaneous injection route of administration and high cost have limited the clinical application of GLP-1R agonists. Renal outcomes have been assessed and compared between these common anti-diabetic medications. SGLT2 inhibitors are known for their protective effects on the kidney in both diabetic and non-diabetic patients (Palmer and Clegg, 2023). Furthermore, SGLT2 inhibitors, GLP-1 analogs, and metformin have been shown to be associated with a lower risk of adverse renal outcomes compared with other types of anti-diabetic medications (Xie et al., 2020; Hung et al., 2023).

Of note, two types of thermogenic adipose tissue have been proven to exist in humans: brown adipose tissue (BAT) and beige adipose tissue. Unlike WAT, thermogenic adipose tissue could convert surplus energy into heat by mitochondria through the mechanism of so-called non-shivering thermogenesis, contributing to energy homeostasis. Classic mechanisms of non-shivering thermogenesis include stimulation of β-adrenergic receptors (β-ARs) by norepinephrine (NE), which is released from the sympathetic nerve terminal, and UCP1-mediated heat generation through mitochondria. In particular, beige adipose tissue is heterogeneous with high plasticity and could emerge in the depot of WAT. They could be most potentially targeted for the treatment of obesity, and thus, obesity-related diabetes. Human BAT is mainly located in the cervical, supraclavicular, paravertebral, and periadrenal areas, according to previous human studies (Nedergaard et al., 2007). Notably, the activity of BAT in humans is negatively correlated with body mass index (BMI) and positively correlated with glucose tolerance and insulin sensitivity (Matsushita et al., 2014). It has been subsequently indicated that BAT also exists in the human perirenal adipose tissue depot but with a broad difference among individuals (Svensson et al., 2014). It mainly consists of unilocular adipocytes with gene expression partially overlapping with either periadrenal multilocular adipocytes (UCP1, etc.) or subcutaneous WAT, which differ in their morphology from the cells near the adrenal gland (Jespersen et al., 2019). However, the function of perirenal BAT is poorly described.

**FIGURE 1:**
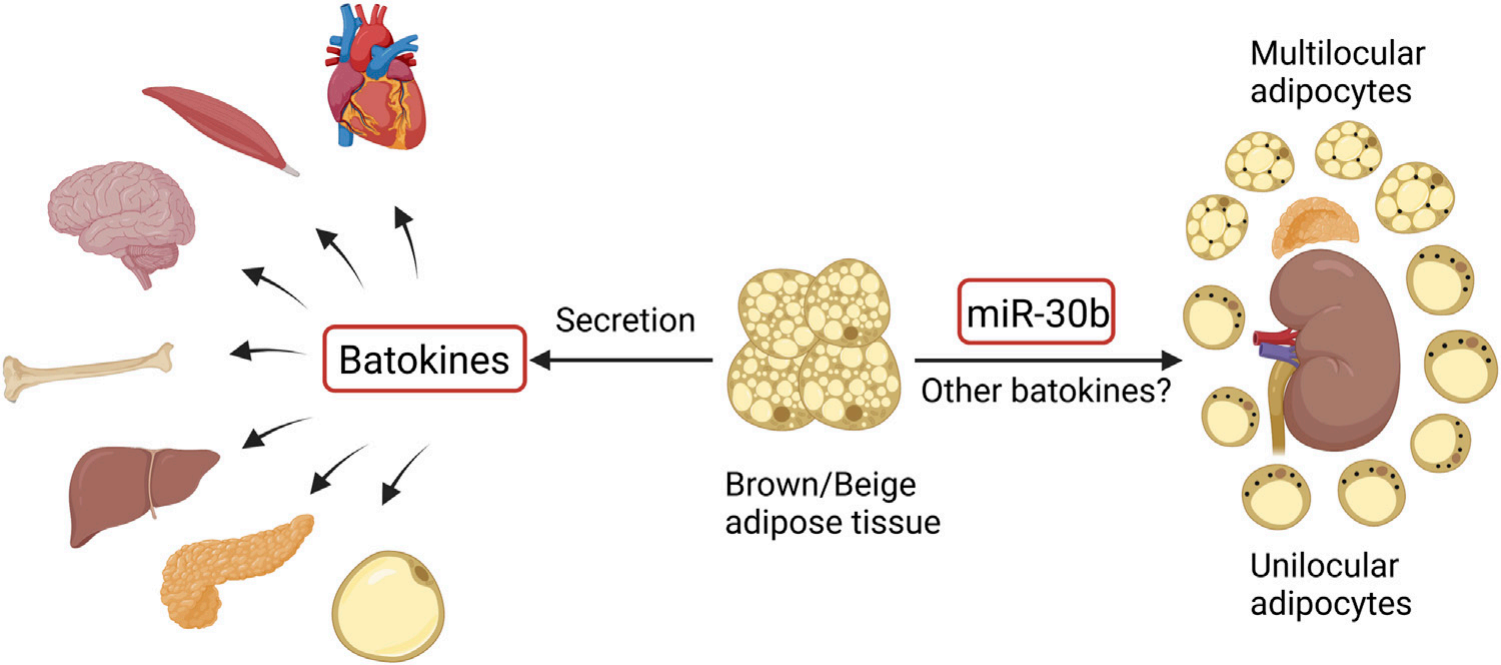
Crosstalk between brown/beige adipose tissue and distant organs and regulation of perirenal BAT (black dots in perirenal adipocytes represent UCP1). The figure was created using BioRender (https://biorender.com/).

In addition to being a thermogenic organ, brown and beige adipose tissue is also recognized as an endocrine organ, involved in the regulation of distant organs through the secretion of factors and microRNAs, which are termed “batokines” (Figure 1). So far, these distant organs include the heart, skeletal muscle, brain, bone, liver, pancreas, and WAT, as summarized by several groups (Thomou et al., 2017; Villarroya et al., 2019; Ahmad et al., 2021). In recent years, a crosstalk between BAT and the kidney has also been described (Figure 1). It has been demonstrated that the browning of renal peritumoral adipose tissue stimulates the expression of epithelial–mesenchymal transition markers in human renal cells (Ferrando et al., 2022), suggesting a detrimental aspect of the perirenal adipose tissue that potentially contributes to tumor development and metastasis. Nevertheless, BAT activation shows metabolic benefits and renoprotective effects in diabetic mice (Cai et al., 2019). Particularly, transplantation of BAT from normal mice to diabetic mice near the interscapular region significantly ameliorates diabetic kidney disease (on the aspects of renal dysfunction, inflammation, oxidative stress, and fibrosis) by downregulating the nephropathy-related genes *Runx1* and *Snail1* in BAT-derived microRNA-30b in diabetic kidneys, a telecrine effect of BAT (Zhang et al., 2021). Massive investigations are required to further explore the function of perirenal BAT and the regulatory mechanisms of possible BAT–kidney crosstalk.

Various anti-diabetic medications have thus been reassessed to investigate whether they play a role in the regulation of thermogenic adipose tissue. In this review, we summarize findings on the thermogenic effects of certain anti-diabetic medications commonly used in the clinic, and potential receptors in adipose tissue that could be targeted to combat obesity, providing novel viewpoints in the intervention and treatment of obesity-related diabetes and possibly diabetic complications (such as diabetic kidney disease).

## 2 Common anti-diabetic medications that may play a role in regulating thermogenic adipose tissue

**FIGURE 2:**
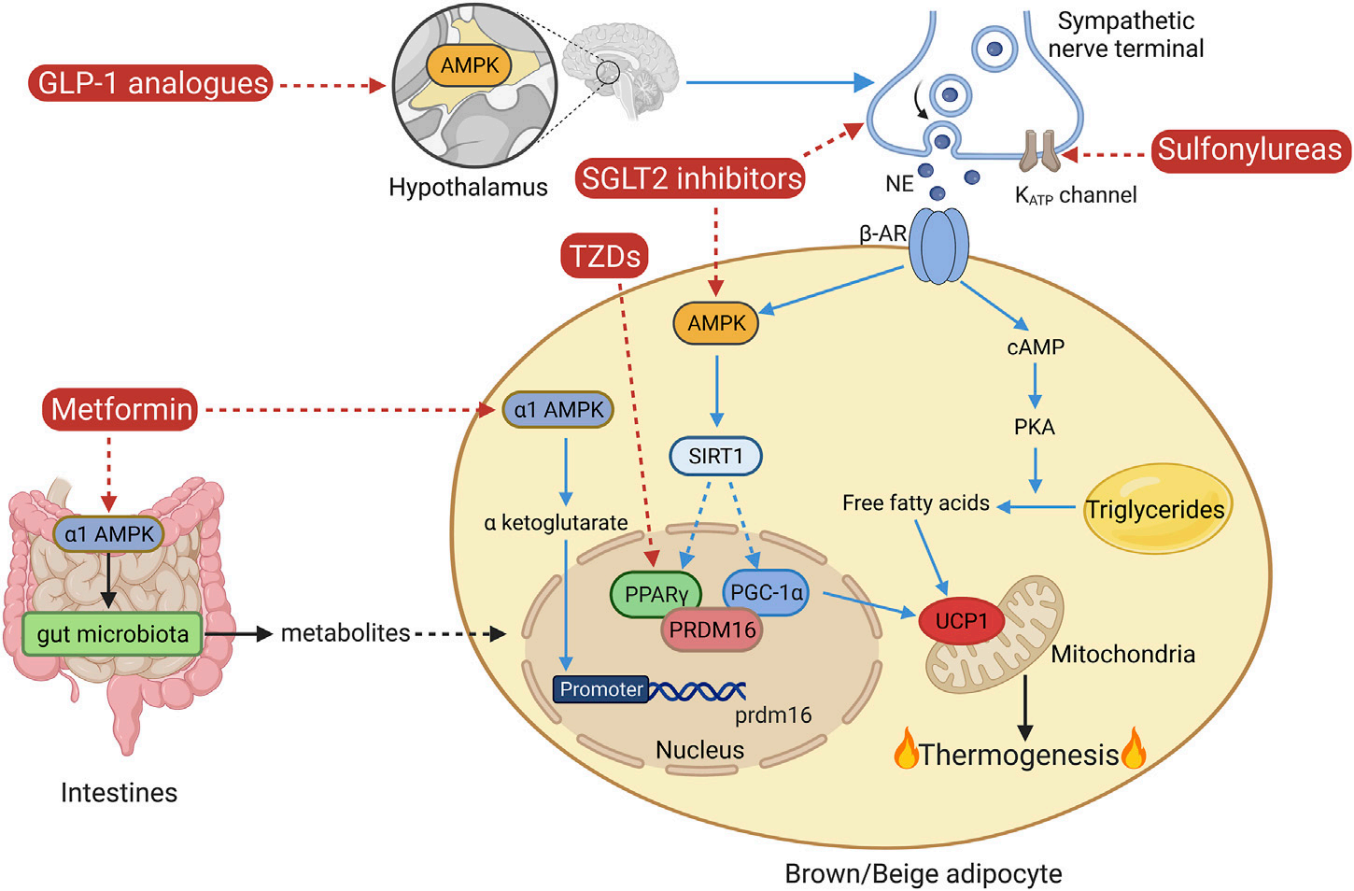
Classical non-shivering thermogenesis and the mechanisms of certain anti-diabetic medications. AMPK, AMP-activated protein kinase; AR, adrenergic receptor; GLP-1, glucagon-like peptide-1; NE, norepinephrine; PKA, protein kinase A; PPARγ, peroxisome proliferator-activated receptor gamma; PGC-1α, PPARγ coactivator-1α; PRDM16, protein PR domain-containing 16; SGLT2, sodium-glucose co-transporter 2; SIRT1, sirtuin 1; TZDs, thiazolidinediones; UCP1, uncoupling protein 1. The figure was created using BioRender (https://biorender.com/).

Owing to the potential of brown and beige adipose tissue in combating obesity, which remains the leading cause of T2DM, targeting these thermogenic adipose tissues may be theoretically and practically feasible to benefit and ameliorate obesity-related diabetes. Along with the increasing knowledge of these two types of thermogenic adipose tissue over the decades, it is questionable whether the medications clinically used for T2DM treatment also play a role in the regulation of brown/beige adipose tissue-mediated thermogenesis. Thus, certain anti-diabetic medications have been reassessed for their effects on thermogenesis. Certain types of anti-diabetic medications have been reported to potentially regulate thermogenic adipose tissue. They include sulfonylureas, TZDs, metformin, SGLT2 inhibitors, and GLP-1 analogs, as shown in Figure 2.

### 2.1 Sulfonylureas

Sulfonylureas have been found to lower blood glucose by stimulating the release of insulin from pancreatic β-cells, inhibiting glucagon secretion from pancreatic α-cells, directly acting on the liver to reduce the clearance rate of insulin and glycogenolysis, and enhancing the sensitivity of peripheral tissues to insulin (Lv et al., 2020). Specifically, the actions of sulfonylureas on pancreatic β-cells include binding with their receptors, which are tightly linked to ATP-sensitive potassium (K_ATP_) channels, inhibiting the activity of K_ATP_ channels, whose closure is related to calcium ion flow into the cells and thus insulin secretion (Ashcroft, 2000; Mikhailov et al., 2000). Furthermore, sulfonylureas activate cyclic adenosine monophosphate (cAMP), and stimulate the translocation of exchange protein2 activated by cAMP (Epac2A) to the membrane, which is a protein involved in the cAMP–protein kinase A (PKA) pathway, or directly activate Epac2A/Rap1 signaling by binding to Epac2A, which regulates the calcium kinetics to enhance insulin secretion (Shibasaki et al., 2007; Seino et al., 2017; Robichaux and Cheng, 2018). The actions of sulfonylureas on pancreatic α-cells include a direct inhibition of glucagon secretion by sulfonylureas and an indirect inhibition of glucagon secretion by sulfonylurea-induced insulin from adjacent β-cells in a paracrine manner (Landstedt-Hallin et al., 1999; ter Braak et al., 2002). Direct effects of sulfonylureas on adipose tissue may include inhibition of lipolysis and triglyceride lipase and promotion of glucose uptake and oxidation (Sola et al., 2015). One of the sulfonylureas, glibenclamide, has been found to improve high-fat diet (HFD)-induced depressive-like symptoms in mice by restoring K_ATP_ channel expression on the surface of brown adipocytes, which is accompanied by improved sympathetic innervation of interscapular BAT (involvement of K_ATP_ channel closure, sympathetic nerve depolarization, and promotion of NE release) and activation of β3 AR-PKA signaling (Kuo et al., 2020). However, in this study, they did not show any direct evidence of improved activation of BAT or enhanced thermogenesis and energy expenditure induced by glibenclamide, though an improved sympathetic innervation of BAT may indicate these effects. Further investigations are thus required in this aspect.

### 2.2 TZDs (PPARγ activators)

PPARγ is a nuclear receptor that is highly expressed in adipose tissue along with other tissues in the colon and placenta (Rosen and Spiegelman, 2000). It plays an important role in the regulation of lipid and glucose metabolism in concert with PPARγ coactivator-1α (PGC-1α), fulfilling the oxidation of fatty acids in mitochondria and maintaining insulin sensitivity in skeletal muscle, adipose tissue, and liver (Hevener et al., 2003; Alaynick, 2008). Thus, PPARγ activation in adipose tissue results in the induction of adipogenesis, lipid metabolism, and promotion of glucose homeostasis (Ahmadian et al., 2013). TZDs are synthetic PPARγ activators designed to improve insulin sensitivity in patients with T2DM. PPARγ, as one of the key factors regulating brown and beige adipogenesis, mediates the effect of TZDs on brown and beige adipose tissue. Indeed, different TZDs, such as lobeglitazone, rosiglitazone, and pioglitazone, show different efficiencies in the regulation of thermogenesis and glycometabolism (Sohn et al., 2018). Rosiglitazone has been found to induce beige fat development in mice probably through the activation of the sirtuin 1 (SIRT1)–PR domain-containing protein 16 (PRDM16) pathway (Ohno et al., 2012; Qiang et al., 2012). Despite inducing beige adipose tissue development, certain TZDs, like pioglitazone, have also been characterized by a weight gain effect. Evidence shows that pioglitazone significantly reduces the activation of BAT in response to cold in humans, which may partially explain the weight gain effect of pioglitazone (Loh et al., 2018). In addition, the full agonism of PPARγ by TZDs is associated with various side effects. Partial or non-TZD PPARγ agonism may reduce side effects while treating metabolic disorders with PPARγ activators. Hence, a novel non-TZD partial PPARγ activator, which is an indole-based chemical and inhibits the phosphorylation of PPARγ, WO95E, has recently been developed with minimal side effects (Wu et al., 2021). It not only ameliorates insulin resistance and glucose tolerance in HFD-induced obese mice but also induces beiging in the inguinal WAT of mice and increases energy expenditure to counter obesity. It also shows beneficial effects on adipose tissue inflammation and obesity-associated liver steatosis. Thus, potential PPARγ activators with minimal side effects are desired to accomplish the treatment of obesity and related diabetes by targeting thermogenic adipose tissue.

### 2.3 Metformin

Metformin, as a potent AMP-activated protein kinase (AMPK) activator, is a first-line anti-diabetic medication and plays a very important role in glucose metabolism and energy homeostasis. It targets various organs and exerts metabolic effects. In adipose tissue, it promotes glucose uptake and attenuates fat accumulation and inflammation through AMPK activation and modulation of other factors (FGF21, mTOR, etc.) (Ziqubu et al., 2023). In addition, metformin has been found to promote thermogenesis and have an anti-obesity effect. Its treatment in mice led to an increase in BAT mass, an induction of beige adipocytes in inguinal WAT, and a probable promotion in adipocyte proliferation and differentiation, in addition to an enhancement in the expression of various thermogenic markers in BAT, which was probably accompanied by improved mitochondrial biogenesis, lipolysis, and fatty acid uptake in BAT (Karise et al., 2019). Introduction of metformin through dissolvable poly-microneedles to the subcutaneous WAT of obese mice with the assistance of iontophoresis led to a marked beiging in inguinal WAT, an increase in energy expenditure, a decrease in body weight and fat mass, and thus an improvement in energy metabolism (Abbasi et al., 2022). Evidence shows that the AMPK α1 isoform mediates the DNA demethylation in the promoter of *Prdm16*, which is very essential for brown adipogenesis, through a key metabolite of the Krebs cycle, α-ketoglutarate (Yang et al., 2016). In addition, Zhang E. et al. (2022) revealed that the effects of metformin on BAT may also include intestine–BAT communication through intestinal α1AMPK-mediated regulation of gut microbiota and their metabolites. Thus, the therapeutic effect of metformin on diabetes may be partially dependent on its acting on brown and beige adipose tissue through various mechanisms.

### 2.4 SGLT2 inhibitors

SGLT2 inhibitors work as anti-diabetic medications through the inhibition of glucose reabsorption through the renal tubules, which could reduce blood glucose by increasing glucosuria. A weight loss effect of SGLT2 inhibitors has been observed; however, it is less than expected based on the energy excretion via glucosuria. Evidence shows that certain SGLT2 inhibitors, such as canagliflozin, ipragliflozin, and empagliflozin, increase energy expenditure and thus ameliorate diet-induced metabolic changes. They promote thermogenesis via inducing beiging in subcutaneous WAT, protect mice from HFD-induced fat accumulation in BAT, enhance mitochondrial biogenesis and respiratory activity, and promote lipolysis and fatty acid oxidation in adipose tissue to varying degrees (Lee et al., 2021; Xu et al., 2021; Yang et al., 2021). The mechanisms of the aforementioned regulation may involve the activation of AMPK and therefore its downstream factors, SIRT1 and PGC-1α, and elevation of the sympathetic innervation of adipose tissue, which facilitates fat mobilization via the β3-adrenergic receptor (β3AR)–cAMP–PKA signaling pathway. Thus, the beneficial effects of SGLT2 inhibition in diabetes may also include its various regulatory effects on adipose tissue.

### 2.5 GLP-1 analogs

GLP-1 analogs, also known as GLP-1R agonists, have become popular anti-diabetic drugs in recent years because of their additional anti-obesity effect. Administration of one of the GLP-1 analogs, semaglutide, to obese diabetic patients, results in a significant reduction in their weight, BMI, fasting glucose levels, and hemoglobin A1c levels without affecting the skeletal muscle mass (Ozeki et al., 2022). Several studies were performed over the years to clarify whether there are other mechanisms by which GLP-1Rs regulate energy metabolism in addition to suppression of appetite and adipose/hepatic lipogenesis and promotion of insulin secretion and sensitivity. GLP-1R activation led to the induction of interscapular BAT thermogenesis with an improved sympathetic innervation. However, GLP-1R signaling is not required for cold-induced thermogenesis since GLP-1R-knockout mice display a normal thermogenic response to cold exposure, suggesting that induction of BAT thermogenesis may be only one of the metabolic effects of GLP-1R signaling on metabolic regulation (Lockie et al., 2012). Similarly, GLP-1R signaling is not required for NE-stimulated oxygen consumption in BAT in mice fed with a chow diet; however, it impacts NE-induced increases in BAT oxygen consumption in mice fed with HFD (Heppner et al., 2015). In addition, chronic administration of the GLP-1R agonist, liraglutide (even at a higher dose, 30 nmol/kg per day), is unable to activate BAT in the presence of GLP-1R in diet-induced obese mice (Heppner et al., 2015), which further indicates that GLP-1R signaling is not indispensable for the BAT-related weight-lowering effect of liraglutide. Ulteriorly, it was demonstrated that central injection of the GLP-1R agonist, liraglutide, in mice could stimulate thermogenesis in BAT and beiging in WAT by activating AMPK in the hypothalamic ventromedial nucleus (Beiroa et al., 2014). Kooijman et al. (2015) observed similar effects and improved glycolipid metabolism after central administration of another GLP-1 analog, exendin-4, suggesting that the beneficial effects of GLP-1 signaling activation on BAT and WAT are probably mediated by enhancing sympathetic innervation of these adipose tissues. Nevertheless, a protective effect of exendin-4 on diabetic kidneys has been suggested to be independent of BAT activation (Fang et al., 2020). Further investigations are required to clarify the regulatory mechanisms of GLP-1 signaling intervention in the treatment of obesity and diabetes.

## 3 Targetable receptors and their potential

Despite the thermogenic effects of the aforementioned anti-diabetic medications, the regulatory mechanisms of these medications on adipose tissue are unclear. In addition, the contribution of these medications to combating obesity and obesity-related diabetes by targeting brown and beige adipose tissue is very limited. Due to the strong energy dissipation capability of these thermogenic adipose tissues, the regulatory mechanisms involved in evoking their activity have recently attracted great interest. Notably, receptors in adipose tissue could be readily targeted by their agonists or antagonists. Here, we also highlight several prominent receptors in adipose tissue that are involved in the regulation of non-shivering thermogenesis and could be potentially targeted to achieve efficient energy dissipation.

### 3.1 β2/β3-adrenergic receptors and A_2A_/A_2B_ adenosine receptors

Several promising receptor signaling pathways have been previously summarized in one of our reviews, such as β2/β3-AR and A_2A_/A_2B_ adenosine receptor signaling pathways (Pan et al., 2020; Pan and Chen, 2022). Investigations of these receptor signaling pathways including the effects of some competent receptor agonists on fat metabolism, have been gradually performed in human cells and human bodies. Excitingly, the dual-active adenosine receptor ligand LJ-4378, with A_2A_ receptor agonist and A_3_ receptor antagonist activity, showed a stronger lipolytic effect than single-substance administration alone in mice, with significant anti-obesity and glucose-improving effects (Kim et al., 2022). Despite great advancements, there remains bemusement in treating obesity and metabolic disorders by targeting these receptors. Reasons include (1) side effects (such as on the cardiovascular system) caused by a high dosage of β3-AR agonist (mirabegron) that is still effective in increasing lipolysis and energy expenditure; and (2) limited investigations of β2-AR agonists and A_2B_ receptor agonists in humans. Thus, the challenges of these receptor agonisms require us to perform further investigations.

### 3.2 Other PPARs

PPARs play an important role in maintaining whole-body glucose and lipid homeostasis. PPAR isoforms show different tissue distributions, among which PPARγ and PPARβ/δ show similar expression levels in WAT and BAT, while PPARα shows the highest expression in BAT (Gross et al., 2017). Evidence shows that PPARα is indispensable for BAT growth and thus for non-shivering thermogenesis in response to cold, regulating BAT biology probably through its cooperation with PGC-1a and PRDM16 (Barbera et al., 2001; Hondares et al., 2011). The PPARα agonist fenofibrate (clinically used for the treatment of dyslipidemia), has shown a beiging effect on subcutaneous WAT in obese mice, increasing energy expenditure (Rachid et al., 2015a, b). Another selective PPARα agonist, pirinixic acid, has been shown to counter HFD-induced BAT whitening in mice, probably by activating the thermogenic pathway and enhancing lipolysis (Miranda et al., 2020). PPARβ/δ has been shown to activate fatty acid oxidation so that it enhances thermogenesis and prevents obesity, which may involve the participation of PGC-1α. However, its agonists have not yet been used in the clinic. The importance of PPARγ, the most expressive PPAR isoform in adipose tissue, has been shown in the regulation of fat metabolism. Its agonists, TZDs, as insulin sensitizers, are commonly used for the treatment of T2DM, which also fulfills a beiging effect (Ohno et al., 2012; Qiang et al., 2012; Wu et al., 2021). As for the challenges and advancements of PPARγ agonism for the treatment of obesity and T2DM, they have been discussed in the previous section.

### 3.3 Glucocorticoid/mineralocorticoid receptors

Glucocorticoid stimulation has been found to diminish thermogenic capacity with a decreased uncoupling protein 1 (UCP1) protein level and an increased accumulation of lipids in BAT, which has thus been considered to mediate glucocorticoid-induced obesity (Poggioli et al., 2013; Scotney et al., 2017; Thuzar et al., 2018). Importantly, the effects of glucocorticoids on brown adipocytes are dependent on their binding to their receptors, which are glucocorticoid receptors located in cytosolic fractions. The liganded glucocorticoid receptors then transferred to the nucleus, where they could bind to various binding sites on the *ucp1* gene and regulate *ucp1* transcription (Luijten et al., 2019). Among them, glucocorticoid receptors binding to the upstream of the transcription start site of *ucp1* cause inhibition in *ucp1* transcription. Thus, the inhibitory effect of glucocorticoid receptor signaling on thermogenic capacity is probably due to the binding of glucocorticoid receptors to *ucp1* that inhibits *ucp1* transcription (“masking” of UCP1). Accordingly, administration of glucocorticoid receptor antagonists inhibits glucocorticoid receptor activity and contributes to UCP1 “unmasking,” most of which (e.g., CORT125281 and C108297) prevent mice from HFD-induced lipid accumulation in BAT and/or result in a decrease in fat mass and body weight (Kroon et al., 2018; van den Heuvel et al., 2016). Furthermore, mineralocorticoid receptor antagonism by spironolactone, drospirenone, and finerenone also shows positive effects on HFD-induced lipid accumulation in BAT and fat accumulation (Armani et al., 2014; Marzolla et al., 2020). In addition, spironolactone and drospirenone possess a beiging effect in the WAT of HFD mice (Armani et al., 2014). In addition, spironolactone treatment in healthy humans results in an enhancement in BAT function (activity, volume, and supraclavicular skin temperature) in response to cold and an elevation in supraclavicular skin temperature and energy production rate in response to a mixed meal (Thuzar et al., 2019). The use of these antagonists is still limited in clinical practice because of the simultaneous antagonism of androgen and progesterone receptors, which cause parallel clinical symptoms (Lainscak et al., 2015; Luijten et al., 2019). In addition, abnormal renal function is associated with a higher risk of hyperkalemia in patients treated with spironolactone, which could threaten the patients’ lives (Schepkens et al., 2001). Thus, great efforts are required to achieve more research progress in this field.

### 3.4 Thyroid hormone receptors

Thyroid hormones have long been known to increase energy consumption and reduce fat. Early findings revealed that they bind to various thyroid hormone receptors (including α and β isoforms) in adipocytes to promote adaptive thermogenesis through crosstalk between thyroid hormone receptor signaling and β AR signaling (Ribeiro et al., 2001). Lateral ventricular injection of triiodothyronine (T3) led to an increase in sympathetic output and an enhancement in BAT activity and thermogenesis (Lopez et al., 2010). In addition, T3 has also been demonstrated to induce beiging (Martinez-Sanchez et al., 2017). Due to the probable but unexpected adverse effects on the heart, bones, etc., caused by excess thyroid hormones, thyroid hormone analogs have been developed to obtain the beneficial effects of thyroid hormone receptor activation with minimal side effects. As such, pharmacological stimulation of thyroid hormone receptors by their agonist GC-1 (β-isoform-selective) has been shown to markedly promote beiging in WAT and induce adaptive thermogenesis in mice (Lin et al., 2015). Notably, the chemical hybridization of glucagon and thyroid hormone results in the concentration of thyroid hormone in WAT, where high levels of glucagon are expressed, the subsequent induction of beige fat development, and an improvement in energy expenditure (Finan et al., 2016). Despite the prolonged controversy on the thermogenic mechanisms of thyroid hormones, it has been recently revealed that T3-thyroid hormone receptor signaling plays a crucial role in the regulation of energy metabolism and BAT proliferation (Zekri et al., 2022). Moreover, the regulatory effects of T3 on BAT hyperplasia have been shown to be mediated by thyroid hormone receptor α through the promotion of progenitor cell proliferation (Liu et al., 2022). Thus, the development of selective agonists against the α isoform of thyroid hormone receptors and further evaluation of their potential effects on thermogenesis is also required.

### 3.5 Other promising receptors

Several other prominent receptors in adipocytes mediate the stimulatory or inhibitory effects of external signals on energy metabolism. They have been summarized in some of our previous reviews, which include the secretin receptor, G protein-coupled receptor 3, neurotensin receptor 2, fibroblast growth factor receptor 3, interleukin-27 receptor α subunit, androgen receptor, estrogen receptors, and follicle-stimulating hormone receptor (Pan et al., 2020; Pan and Chen, 2022). Recently, the specific roles of some other receptors have also been identified. The interleukin (IL)-18 receptor mediates the IL-18 signaling in WAT to promote insulin sensitivity and glucose uptake, whereas the Na-Cl co-transporter mediates the IL-18 signaling in BAT to enhance thermogenesis (Zhang X. et al., 2022). The nuclear bile acid receptor-farnesoid X receptor has been recently found to negatively regulate BAT development and function in mice (Yang et al., 2023). Hydroxycarboxylic acid receptor 1 in BAT plays an important role in maintaining glucose homeostasis (Min et al., 2022).

## 4 Prospects

The basic treatment of diabetes remains complex, inefficient, and sometimes contradictory, even though more and more anti-diabetic medications are being developed. Especially when patients have complications such as diabetic nephropathy, treatment with anti-diabetic drugs becomes problematic due to renal excretion of most of the medications. Obesity is one of the main causes of T2DM, so combating obesity could potentially be the primary treatment for T2DM and its complications. Due to the potent function of BAT and beige adipose tissue on fat burning and energy expenditure, targeting these thermogenic adipose tissues could be a promising strategy to combat obesity and thus obesity-related diabetes, independent of insulin and glucose levels. In addition, as an endocrine organ, activated thermogenic adipose tissue secretes “batokines” that may be involved in the regulation of distant organs. The crosstalk between these adipose tissues and the kidney has been partially described (Figure 1), and there exist special types of BAT in the perirenal region *per se* (Figure 1). The function of the perirenal BAT and the crosstalk between BAT and the kidney should be further investigated.

Meanwhile, the function of the classic anti-diabetic medications on thermogenic adipose tissue has been studied by many groups. The potential roles of these medications in the regulation of non-shivering thermogenesis are shown in Figure 2. Certain anti-diabetic drugs play well-defined roles in the regulation of thermogenic adipose tissue, such as PPARγ activators, metformin, SGLT2 inhibitors, and GLP-1R agonists, which are also summarized in Table 1. However, investigations of the mechanisms of the effects of these anti-diabetic medications on thermogenic adipose tissue are so far very limited and truly require further research.

**TABLE 1:** Common anti-diabetic medications that play a role in the regulation of thermogenic adipose tissue.

Categories of anti-diabetic medications	Anti-diabetic mechanism	Drugs that act on thermogenic adipose tissue	Thermogenic adipose tissue effects and potential mechanisms	References
Sulfonylureas	Stimulates insulin secretion; reduces insulin clearance and glucagon secretion; improves insulin sensitivity	Glibenclamide	Improves sympathetic innervation of BAT; activation of β3 AR-PKA signaling	Kuo et al. (2020)
PPARγ activators	Improve insulin sensitivity	Lobeglitazone, rosiglitazone, pioglitazone, and WO95E	Induce beiging; activate the SIRT1–PRDM16 pathway	Ohno et al. (2012); Qiang et al. (2012)
Metformin	Activates AMPK; enhances the uptake and usage of glucose	Metformin	Increases BAT mass and expression of thermogenic markers in BAT; improves mitochondria biogenesis, lipolysis, and fatty acid uptake in BAT; activates intestinal α1AMPK and builds an intestine–BAT communication; promotes brown adipogenesis and thermogenesis through αlAMPK–PRDM16 signaling; induces beiging	Yang et al. (2016); Karise et al. (2019); Abbasi et al. (2022); Zhang et al. (2022a)
SGLT2 inhibitors	Inhibit glucose reabsorption through renal tubules	Canagliflozin, ipragliflozin, and empagliflozin	Induce beiging; enhance mitochondrial biogenesis and respiratory activity; promote lipolysis and fatty acid oxidation in adipose tissue; activate AMPK- sirtuin 1- PGC-1α signaling; elevate the sympathetic innervation of adipose tissue; facilitate fat mobilization via β3AR–cAMP–PKA signaling	Lee et al. (2021); Xu et al. (2021); Yang et al. (2021)
GLP-1 analogs	Suppress appetite and delay gastric emptying; facilitate insulin secretion from pancreatic β-cells	Liraglutide and exendin-4	Induce BAT and beige adipose tissue thermogenesis; activate AMPK in the hypothalamic ventromedial nucleus; improve sympathetic innervation of thermogenic adipose tissues	Lockie et al. (2012); Beiroa et al. (2014); Kooijman et al. (2015)

In addition, numerous receptors in adipose tissue have been found to mediate the action of various substances on thermogenic adipose tissue that could theoretically be targeted to induce BAT and beige adipose tissue activity. Main receptors and their actions on thermogenic adipose tissue (when agonized or antagonized) and the limitations of their use have been summarized in Table 2. Progress in the development of receptor agonists or antagonists has been made to minimize the side effects. However, with the exception of PPARγ inhibitors for the treatment of obesity-related diabetes, most of them have not been approved to treat obesity. In addition, thermogenic mechanisms involving other promising receptors are gradually being discovered, although agonists or antagonists are not yet available. Although challenging, further investigations of receptor signaling are required for a significant transition from basic research to clinical practice and better and more effective treatment of obesity-related diabetes.

**TABLE 2:** Targetable receptors in adipose tissue and their actions and limitations.

Receptor	Beneficial action	Actions on thermogenic adipose tissue	Potential mechanism	Reasons for their limitation
β2/β3-Adrenergic receptors	Agonism	Increase BAT activation; induce beiging	Activate cAMP/PKA signaling; co-express with UCP1	Side effects such as cardiovascular dysfunction
A_2A_/A_2B_ adenosine receptors	Agonism	Increase BAT activation and induce beiging	Heterodimerization of A_2B_ and A_2A_ receptors	Uncertain
PPARα/β/γ/δ	Agonism	Regulates BAT biology; induces beiging; counters against HFD-induced BAT whitening	Cooperates with PGC-1α and PRDM16; activates the thermogenic pathway and enhances lipolysis; activates fatty acid oxidation	Weight gain effect; side effects such as hepatotoxicity, myocardial infarction, bladder cancer, and heart failure
Glucocorticoid/mineralocorticoid receptors	Antagonism	Prevent HFD-induced lipid accumulation in BAT, decrease fat mass and body weight; induce beiging; enhance BAT function	UCP1 “unmasking”	Antagonism on androgen and progesterone receptors; risk of hyperkalemia
Thyroid hormone receptors	Agonism	Induce beiging and promote adaptive thermogenesis; induce BAT proliferation	Crosstalk between thyroid hormone receptor signaling and β-adrenergic receptor signaling; promote progenitor cell proliferation	Uncertain
